# Numerical analysis of pressure drop reduction of bubbly flows through hydrophobic microgrooved channels

**DOI:** 10.1038/s41598-023-45260-7

**Published:** 2023-11-01

**Authors:** Javane Javaherchian, Ali Moosavi, Seyed Ali Tabatabaei

**Affiliations:** 1 Center of Excellence in Energy Conversion (CEEC), School of Mechanical Engineering, Sharif University of Technology, Azadi Avenue, Tehran, Iran; 2 Department of Mechanical Engineering, University of Tehran, North Karger Avenue, Tehran, Iran

**Keywords:** Mechanical engineering, Chemical engineering

## Abstract

Due to the high performance of hydrophobic surfaces in pressure drop reduction, they have been proposed for various applications. However, despite the extensive uses of two-phase flows in many industries, the effect of hydrophobic surfaces on the pressure drop reduction of two-phase flows has not been well understood yet. Thus, in the present study, by implementing the phase-field and finite element methods, the bubbly flows as an example of two-phase flows are considered for examining the effect of hydrophobic microgrooved microchannels on the pressure drop reduction of these regimes in the laminar state. We found out that hydrophobic microgrooved surfaces not only can be efficient in the bubbly flow but also can even cause a maximum pressure drop reduction of up to 70%, which is almost 3.5 times higher than in single-phase flow. We also studied the influence of each parameter, such as bubbles volume or length, Reynolds number, capillary number, and their combination on this phenomenon. The pressure drop reduction grows by increasing the volume of the bubbles but decreases by increasing the flow velocity or the surface tension coefficient. The combination of these parameters demonstrated different results in some circumstances.

## Introduction

Since the early 1970s, with the beginning of the energy scarcity crisis and the rapid development of energy consumption, the scientific community has strived to find numerous ways to conserve energy and reduce energy consumption. Therefore, the drag reduction technology that tries to decrease the friction drag between solid and liquid phases has become very important in related industries such as maritime and land transportation, internal pipelines, and microfluidic devices. Overall, the drag reduction methods are divided into three categories of active approaches that apply the external mass, momentum, or energy to the boundary layer for lessening drag; passive approaches that operate by modifying the surface properties like material, shape, and physics^1,2^; and the compound approaches such as utilizing polymer additives and superhydrophobic walls^3^ that can take advantage of two previous procedures. Polymer additives, gas injection, heating and cooling wall, electromotive force, blowing and suction of mass, and wall vibration and deformation are recognized as active techniques^1^. Polymer injection is the most popular method with effective performance among these methods. However, it demands ongoing maintenance and might pollute the environment, like the blowing and suction mass method. Gas injection and flexible vibrant wall methods cannot be controlled easily. Heating and cooling wall and electromotive force strategies consume massive energy^4^. Thus, passive methods like groove and textured surfaces, coating, flexible wall, and hydrophobic and superhydrophobic surfaces have received considerable attention. Among the mentioned techniques, the hydrophobic and superhydrophobic surface method, inspired by nature, shows reliable outcomes in a drag reduction of both the laminar and turbulent flows^5^. The structures of this coating surface entrap the air and reduce the frictional resistance of the liquid–solid interface by replacing it with the gas–solid interface. The hydrophobic and superhydrophobic surfaces also have other characteristics such as self-cleaning^6^, anti-fogging^7^, anti-corrosion^8^, anti-icing^9^, separation^10^, and developing condensation and boiling heat transfer^11^.

Since the contact angle, slip length, hydrostatic pressure, and roughness are crucial factors in hydrophobic and superhydrophobic surface performance, different methods have been proposed to manufacture appropriate surfaces according to their applications. A hydrophobic surface typically has a static contact angle ranging from 90 to 150 degrees. On the other hand, a superhydrophobic surface has a static contact angle greater than 150 degrees, indicating an extremely high resistance to wetting by water. Increasing the contact angle and slip length, which can be adjusted based on the flow conditions, improves the hydrophobic and superhydrophobic efficiency in drag reduction^12^. However, by increasing hydrostatic pressure over the surface, the performance declines substantially due to the transition from the Cassie (drying state) to Wenzel (wetting state)^13^. For instance, the refined surface of PDMS/hydrophobic silica and sanded Teflon can reduce drag maximumly until 25% and 27% with maximum apparent slip length of 70 µm and 20 µm, respectively^14,15^. Also, offsetting of transverse microgrooves causes an increment of slip length^16^. The roughness properties such as shape, sizes, and scale of grooves critically influence drag reduction. It was shown that the random roughness could cause less performance than the ordered roughness^17^. Mohammadi and Floryan examined four groove shapes (rectangle, triangle, trapezoid, and semicircle). They found out that trapezoidal and triangular grooves had the highest and smallest value of drag reduction, and also, using the grooves on the two sides of channel walls resulted in up to four times of drag reduction compared to one side^18^. Li et al. proved that the dovetail groove had better effectiveness than rectangular, trapezoidal, and triangular grooves^19^. Increasing the depth to width ratio helps the pressure drop reduction initially; however, when this ratio becomes greater than 0.4, this parameter does not affect pressure drop reduction. Also, by shortening the distance between two consecutive grooves, the lower pressure drop is gained. Generally, for each case, there is the optimum size for the width, height, and pitch of the grooves in order to reach the maximum pressure drop reduction^19–22^. Both micro and nanostructures can be productive in superhydrophobic surfaces. Nevertheless, their combination, known as hierarchical grooves, demonstrated excellent drag reduction with high durability and an increase of 100% in slip length compared with microscales^23^. In fact, in dual superhydrophobic surfaces, an air layer created by nanostructures and a vortex generated by microstructures act together in order to enhance the slip length^24^, so based on Li et al.’s paper, these surfaces can be employed even in high hydraulic pressures with 92–96% drag reduction that cannot be gained by either use of microscale and nanoscale surfaces alone^25^ and the interesting part is that most of these surfaces are biomimetics and come from natural materials such as rice and lotus leaf, butterfly and cicada wing, gecko foot, shark and fish skin, so forth^26^. For various flow conditions, Hydrophobic and superhydrophobic surfaces can decrease drag properly (for example, up to 40%^27^) not only in the laminar flow but also in the early step of the turbulent regime (up to 30% pressure drop reduction was observed) and by manufacturing optimized roughness with high and stable air fractions, these surfaces can be practical in all ranges of turbulent flows^28^. The more the Reynolds number (*Re*) grows, the more pressure drop reduction lowers in the laminar flows; however, this effect is the reverse in turbulent regimes. The cause is that in the larger *Re* of turbulent flows, small- and large-scale vortical structures become weaker and reduce the shear stress and the momentum transportation^29^. Apart from reaching maximum drag reduction with the lowest cost and complexity, the durability of hydrophobic and superhydrophobic surfaces is also a significant parameter during the time, which means their ability to sustain the air pockets inside the structures for a long time. One of the ways is controlling the temperature near the surface structures^30^. Another way is improving the surface material by offering mechanically stable hydrophobic surfaces. As mentioned, the generation of sufficient dual micro-nano surfaces also can be helpful in the persistence of surfaces^31,32^. Hu et al. invented alternated superhydrophobic and hydrophilic strips to not only keep the air layers but also achieve 77.2% drag reduction^33^. Besides the Newtonian fluid, the hydrophobic surfaces can even raise the pressure drop reduction^34^ (up to 48% in the special case of shear-thickening flow^35^) for non-Newtonian fluids.

The research on implementing superhydrophobic and hydrophobic surfaces in various fields and inventing methods for improving them in the area of drag reduction has been increasingly growing. One of the challenging fields is pressure drop reduction of multiphase flow, which can be helpful in various purposes such as pipelines, the petroleum industry, naval applications, reactors, plastics and paper-making construction, flow boiling and condensing, and biomedical devices. Although few previous studies reported that in some cases, the drag of multiphase flow in hydrophobic surfaces slightly increased or did not show any effect, Stevens et al. mentioned that drag could be reduced about 10% more in a mixture of water and air than in a single liquid^36,37^. It was noted that for bubbly two-phase flow, the ratio of drag reduction in hydrophobic surfaces to hydrophilic surfaces is higher compared to single-phase flow, so superhydrophobic and hydrophobic surfaces are more effective in the bubbly regime. For productiveness, all the conditions should be optimized. Even adding more air will not lead to more drag reduction^38^. Despite mentioned research and others in this area, this phenomenon requires more scrutiny, and there is still room for its investigation. On the other side, recently, microfluidic devices have attracted much attention due to their remarkable properties in biomedical and diagnosis equipment, micromachining technology, logic systems, and separation^39–47^. Thus, in the present study, by applying the phase-field method, we conduct the numerical simulation to determine the effect of hydrophobic microgrooved channels on the laminar bubbly flow and the relevant parameters such as the bubbles’ length, the velocity of flow, and surface tension. It is concluded that hydrophobic microgrooved channels for bubbly flow can be beneficial and, in some cases, even can be better than the inlet single-phase flow in pressure drop reduction.

## Results

### General problem description

**Figure 1:**
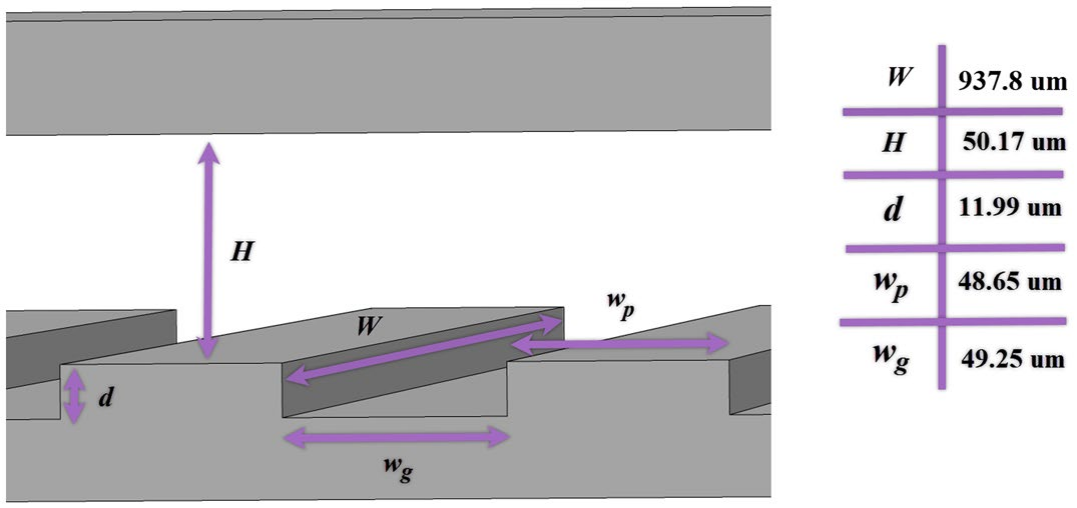
The geometry and dimensions of the hydrophobic microchannel.

The 2D hydrophobic microgrooved channel with defined sizes is considered in all the simulations, as depicted in Fig. 1.

The shape of the microgrooves is a rectangle, and the ratio of width ($W=937.8 \,$µm) to height ($H=50.17 \,$µm) of the channel is more than 18; thus, we can simulate the 3D channel as a 2D channel. The microchannel length of the simulation is assumed to be ${L}_{SIM}=440.25\,$ µm with four microgrooves to minimize the computational time; because, in the validation part, considering one, two, four, eight, and larger numbers of grooves, it is confirmed that the channel with four grooves is a rational choice, and the pressure drop does not change almost.

For selecting the number of grids in each simulation, based on the results of the mesh independence of the validation part, the correct mesh size is determined and considered for each case.

After forming and developing the bubbly flow with various volumes, velocities, and surface tension in a hydrophilic microchannel, it enters the hydrophobic microgrooves channel. Thus, in the first step, the effect of bubbles volume or length in bubbly flow on pressure drop reduction is studied. After that, the flow with constant volume but with several velocities of gas and liquid is simulated to observe the procedure of changing the pressure drop reduction. In the second step, the influence of mentioned parameters is analyzed simultaneously. In the final step, after identifying the two cases with the maximum pressure drop reduction, the variation of pressure drop reduction with capillary numbers is measured in these two states, and the two-phase flow with optimum Reynolds number and capillary number that has the maximum pressure drop reduction is compared with the single-phase flow in the same condition.

### Validation

**Figure 2:**
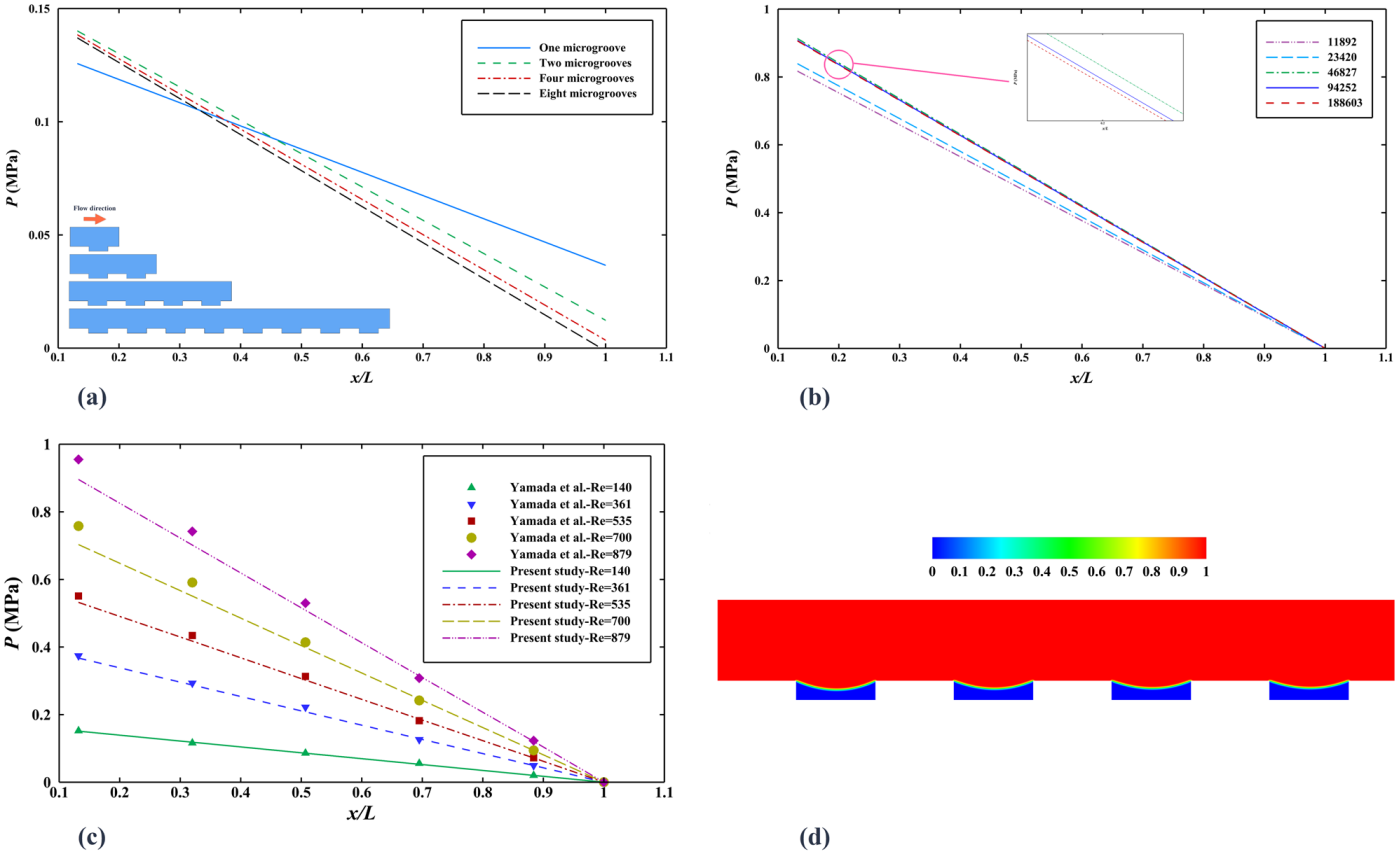
(**a**) The pressure drop in the different lengths of the microchannel (one, two, four, and eight microgrooves) for ${\mathrm{Re}}_{l}$=140. (**b**) The pressure drop for the different numbers of mesh ${\mathrm{Re}}_{l}$=879. (**c**) The pressure drop of experiment^49^ and simulation studies along the microchannel for the five Reynolds numbers. (**d**) The contour of the volume fraction of water for ${\mathrm{Re}}_{l}$=140.

The experimental article presented by Yamada et al.^48^ is supposed to check the accuracy of using the phase-field method. It is assumed that water flow comes into the hydrophobic microgrooved channel that is filled with air. The density and dynamic viscosity of air and water are considered to be at 20 °C. To decrease the computational cost, the ordered rectangular rib-patterned microchannel (Rib-50) with defined sizes and surface wettability is investigated as a 2D geometry since the 2D and 3D pressure drop reduction results demonstrate this assumption’s correctness. Also, the microchannel length is considered to be 420 µm with four microgrooves instead of *L* = 26.65 mm since the simulations confirm that the pressure drop changes linearly through the channel and can be estimated easily. Also, as presented in Fig. 2a, checking the microgroove dependency for Reynolds number of 140 shows that increasing the microchannel length of more than four microgrooves does not change the pressure drop substantially (less than 2% difference) and with four microgrooves and less computational cost, the same result with high accuracy can be achieved. The exact mesh sizes are achieved by initially considering 11,892 triangular grids for the case with a Reynolds number of 879 and increasing mesh numbers until the pressure drop becomes approximately constant. Figure 2b compares pressure drops along the microchannel length based on the number of grids in each simulation. As illustrated in Fig. 2b, the grids numbers of 94,252 cause the acceptable precision with mesh independence in this case and another case (${\mathrm{Re}}_{l}$=140), so the average mesh size of 0.752 µm is acceptable to be applied for the considered domain in all simulations.

Figure 2c shows a comparison between the experimental results of pressure drop along the microchannel from Yamada et al.’s article^49^ and the numerical simulations of fully developed laminar flow with ${\mathrm{Re}}_{l}$=140, 361, 535, 700, 879 that is stable during the time in the hydrophobic microchannel. The average error percentage is about 4%, demonstrating that the method and assumptions are suitable and correct for this system. The interface between the trapped air and water in microgrooves remains constant in the Cassie-Baxter state after a while, and the hydrophobic microchannel works appropriately in this way, as is represented in Fig. 2d.

### Effect of bubbles size and velocity on pressure drop reduction

**Figure 3:**
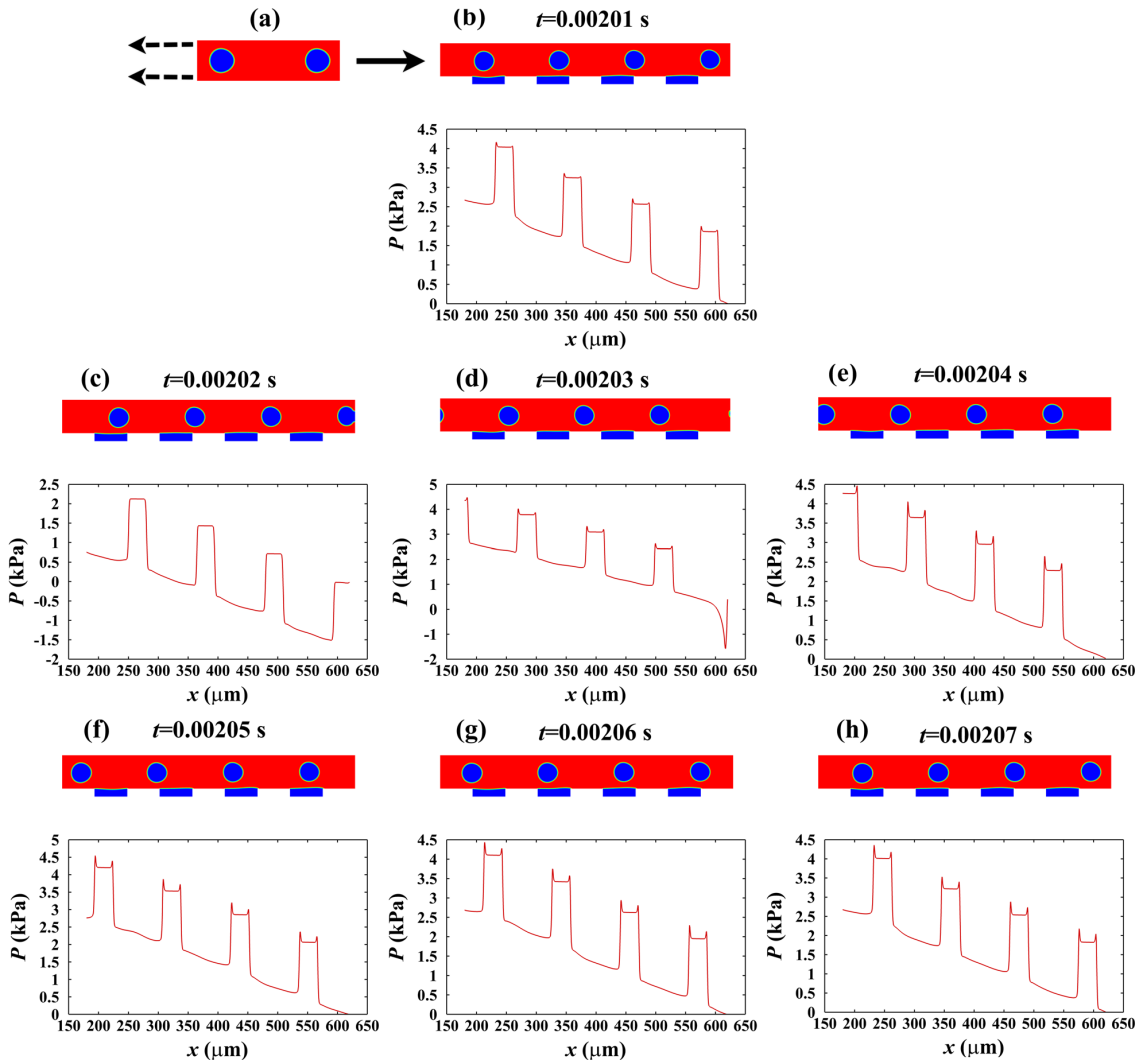
The volume fraction of water and pressure drop along the channel when ${\mathrm{Re}}_{l}=140$ and $Ca=0.06$ for *t* = (**b**) 0.00201 s, (**c**) 0.00202 s, (**d**) 0.00203 s, (e) 0.00204 s, (**f**) 0.00205 s, (**g**) 0.00206 s, (**h**) 0.00207 s.

The hydrophobic microgrooved channel of the validation study is assumed in which the bubbly regime flows from a hydrophilic microchannel. Since we want to study the effect of hydrophobic surfaces on the two-phase regime, the bubbly flow should be stable. Thus, the bubbles and liquid bubbly approach to a hydrophilic microchannel with the same time step and move enough along the channel length to reach its constant shape after the amount of time as displayed in Fig. 3a for the condition when ${\mathrm{Re}}_{l}=140$ and $Ca=0.06$. Then, they move into a hydrophobic microchannel, and after a while, patterns of bubbly flow and trapped air are repeated depending on the frequency of the bubbles unit^50^. As evident, the contour of the volume fraction of water and chart of pressure drop in the middle of the microchannel for Fig. 3b,h are almost similar. Therefore, the average pressure difference between the inlet and outlet of the hydrophobic microgrooved channel in each period (see Fig. 3b,c,d,e,f,g, from 0.00201 s to 0.00206 s) can be gained for each circumstance. The average pressure drop also can be measured with this method when the bubbly regime transfers across the hydrophilic microchannel in the same length (${L}_{SIM}=440.25\,$µm). Therefore, the pressure drop reduction (%PDR) can be determined from Eq. (16) for each regime. In the case of Fig. 3, %PDR is 15.4% which corroborates the usefulness of hydrophobic surfaces for two-phase regimes.

1 $$ \% {\text{PDR}} = \frac{{\left| {{\text{pressure }}\,{\text{drop }}\,{\text{in}}\,{\text{ a}}\, {\text{hydrophobic}}\, {\text{microchannel}} - {\text{pressure }}\,{\text{drop}}\,{\text{ in}} \,{\text{a}} \,{\text{hydrophilic}} \,{\text{microchannel}}} \right|}}{{{\text{pressure}} \,{\text{drop}}\, {\text{in }}\,{\text{a}}\, {\text{hydrophilic}}\, {\text{channel}}}} \times 100 $$

As outlined in Fig. 3, unlike single-phase flow, the pressure drop in two-phase flow consists of the viscous term, which appears due to friction of walls, and the Laplace term due to surface tension at the interface gas–liquid. The oscillations in pressure charts manifest the phase of the bubble and liquid in each cross-section. The higher pressure levels belong to the gas bubbles, and the lower ones display the liquid bubbly section. Taking a careful look at the bubbles in the bubbly flow reveals that the front of the bubbles has a smaller radius than their back because the pressure difference between the liquid and back of the bubbles is lower than the liquid and front of the bubbles, as depicted in Fig. 3. This phenomenon can be seen better in the following.

The bubbles volume or length and velocity of phases can play essential roles in the amount of pressure drop reduction. Thus, by keeping the velocity and capillary constant (${\mathrm{Re}}_{l}=535$ and $Ca=0.06$) and changing the bubbles length or surface area in 2D simulations, the average values of pressure drop reduction are reported in Table 1.

**Table 1:** The bubbly regime at the inlet with different lengths or surfaces and hydrophobic efficiency.

Bubbly regime	The surface area of the inlet bubble (µm^2^)	The ratio of the air bubbles length to the channel height	%PDR
A	1765.0	1.16	30.4
B	2601.4	1.63	31.8
C	3507.1	2.03	36.2%

**Figure 4:**
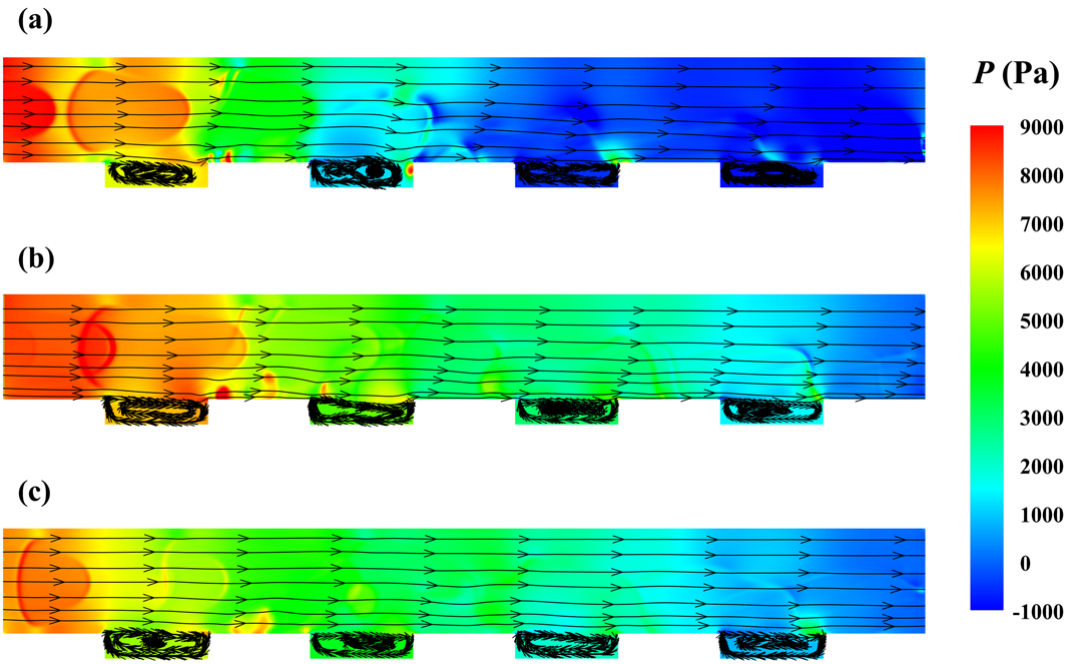
The average pressure distribution with streamline in the period for the case (**a**) A, (**b**) B, (**c**) C.

With the increase of the size of the bubbles, the pressure drop reduction is developed because the regime mixes with more air, replaces more water in microgrooves, and limits the contact surface between the solid surface and water. According to Table 1, the amount of development in pressure drop reduction increases by growing the bubbles size from case A to C since the effect of mixing between bubbles and air pockets in hydrophobic microgrooves develops from A to C. Also, the pressure drop decreases more by shifting from A to C, as Fig. 4a,b,c illustrate the distribution of average pressure with streamlines in a repeatable period for A, B, and C.

**Figure 5:**
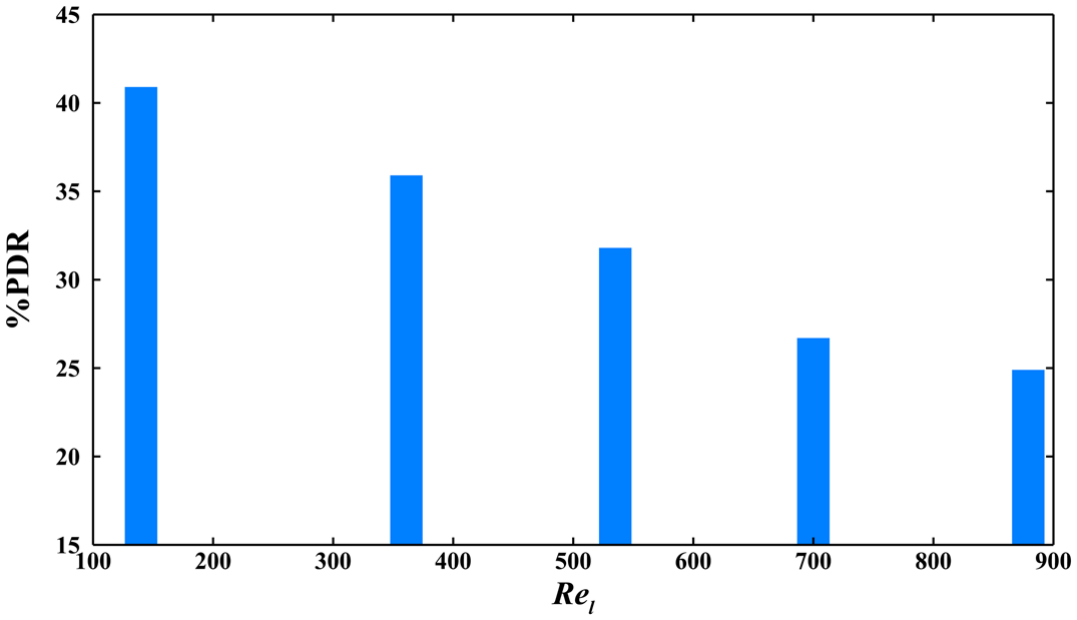
The pressure drop reduction of the constant size (2617.1 µm^2^) of bubbles in bubbly regimes in hydrophobic microgrooved channel for various Reynolds numbers and the capillary number of 0.06.

To understand the influence of velocity on pressure drop reduction, the average periodic pressure drop of the almost constant surface area of the bubbles in a hydrophobic microgrooved channel is compared with it in the hydrophilic channel for several ${\mathrm{Re}}_{l}$. Figure 5 indicates that, as expected, by increasing the Reynolds number, the pressure drop reduction is detracted for the condition that the surface area of the bubbles in bubbly flow is about 2617.1 µm^2^, and the capillary number equals 0.06. As the previous literature expressed, pressure drop reduction of the laminar single-phase flow entering the hydrophobic microchannel decreases by increasing Reynolds number^35^, and the same consequence happens for the two-phase flow.

### Bubbly regimes with various Reynolds numbers

The bubbly regimes with the same constant period and different Reynolds numbers are simulated to investigate the influence of two mentioned parameters simultaneously, namely, bubbles size and velocity. Thus, by enhancing ${\mathrm{Re}}_{l}$ besides the flow velocity, the bubbles surface area or length increases, as is obvious in Table 2, which presents the inlet bubbly regime for each circumstance with a constant capillary number of 0.06 and gas phase sizes in each case.

**Table 2:** The inlet bubbles surface area and length for ${Re}_{l}$ of 140, 361, 535, 700, and 879 and Ca of 0.06.

Bubbly regime	Bubbles surface area of the inlet (µm^2^)	The ratio of bubbles length to the channel height
	693.7	0.60
	1721.6	1.10
	2545.6	1.63
	3396.3	2.13
	4275.6	2.71

**Figure 6:**
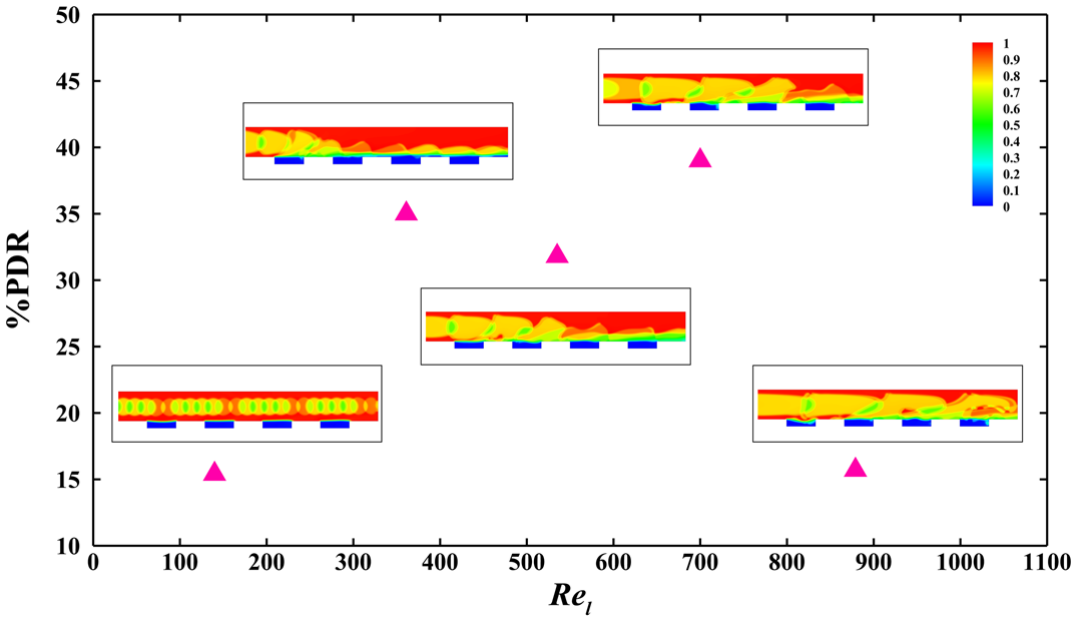
The average volume fraction contours in the same period and the pressure drop reduction for various ${\mathrm{Re}}_{l}$ of 140, 361, 535, 700, and 879 and *Ca* of 0.06.

Figure 6 shows the contour of water volume fraction and amount of pressure drop reduction for each case.

As it is observed, by growing ${\mathrm{Re}}_{l}$, the increase of velocity flow causes the decrease of pressure drop reduction. On the other hand, the growth of bubbly flow sizes leads to the development of pressure drop reduction. Besides these effects, when mixing between bubbly flow and air pocket in microgrooved areas creates, and a part of bubbles enters the microgrooves, the pressure drop reduction promotes due to the more air contact to the surface than liquid as the comparison between two contours of average volume fraction in the period for the cases with ${\mathrm{Re}}_{l}$ of 140 and 361 demonstrates. After that, from ${\mathrm{Re}}_{l}$ of 361 to 535, the pressure drop reduction decreases due to the more substantial effect of increment of velocity than enhancement of bubbles size. However, from ${\mathrm{Re}}_{l}$ of 535 to 700, this fact is reversed, and pressure drop reduction is developed. Finally changing ${\mathrm{Re}}_{l}$ from 700 to 879, and the privilege of velocity flow influence returns the condition to the previous state (from ${\mathrm{Re}}_{l}$ of 535 to 700), and pressure drop reduction is reduced, as specified in the chart of Fig. 6. The average distribution of water volume fractions in this figure and the change of the air amount in microgrooves in each case also affirm this analysis and results.

Consequently, among considered cases, when mixing bubbly regime and air in hydrophobic microgrooves occurs, changing the velocity has more effect on pressure drop reduction than the bubbles length or surface area. However, between ${\mathrm{Re}}_{l}$ of 535 and 879, the effect of bubble size is higher than velocity flow one, which can be seen in ${\mathrm{Re}}_{l}$ of 700.

### Bubbly regimes with different capillary numbers

**Figure 7:**
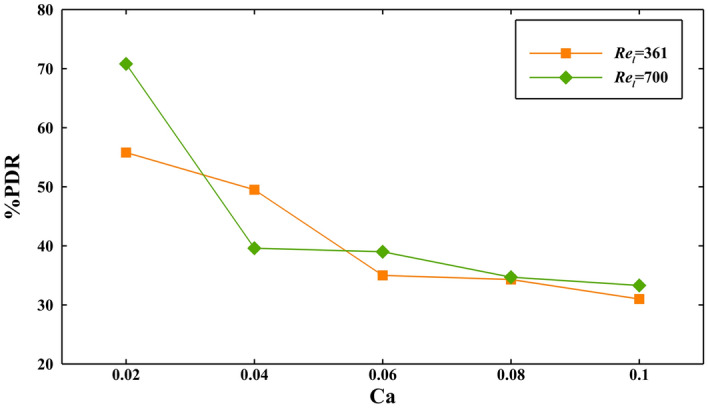
The pressure drop reduction for ${\mathrm{Re}}_{l}=$ 361 and 700 for various capillary numbers.

Among the investigated cases in the previous section, the cases of the bubbly regime with a bubbles size of 3396.3 µm^2^ and ${\mathrm{Re}}_{l}$ of 700 and bubbly regime with a bubbles size of 1721.6 µm^2^ and ${\mathrm{Re}}_{l}$ of 361 in hydrophobic microchannels have almost the most efficiency of 39% and 35%, respectively, in *Ca* of 0.06. To find the optimum condition in which the hydrophobic surface has the most effectiveness, these two cases are supposed and evaluated with different capillary numbers (0.02, 0.04, 0.06, 0.08, and 0.1), as Fig. 7 represents.

For ${\mathrm{Re}}_{l}=700$, the case with *Ca* = 0.02 has the highest pressure drop reduction (70.8%). This is even larger than that for the case with ${\mathrm{Re}}_{l}=361$ and *Ca* = 0.02 (55.8%). In both cases, by increasing the capillary number, the pressure drop reduction decreases because of reducing the surface tension. From *Ca* of 0.06 to 0.1, the change of pressure drop reduction is smaller since the variation of surface tension coefficient is diminished with an increase of *Ca*. Also, it seems that the effect of Reynolds number and velocity of flow is weakened by increasing *Ca* and the ${Re}_{l}$ of 361 and 700 have almost the close amount of pressure drop reduction in *Ca* = 0.06, 0.08, and 0.1. For each capillary number, ${\mathrm{Re}}_{l}$ of 700 has the more pressure drop reduction than ${Re}_{l}$ of 361 except in *Ca* = 0.04. It can be because of this point that in cases with *Ca* of 0.04, the mixing of the bubbly regime with air in microgrooves and hydrophobic characteristics are more efficient in lower Reynolds numbers compared to the cases with *Ca* of 0.02, 0.06, 0.08, and 0.1. Therefore, the combination of all parameters like bubbles size, the velocity of flow, and the surface tension coefficient determines the performance of hydrophobic surfaces in the two-phase flow. They can be optimized so that a maximum pressure drop reduction happens in a given condition.

**Figure 8:**
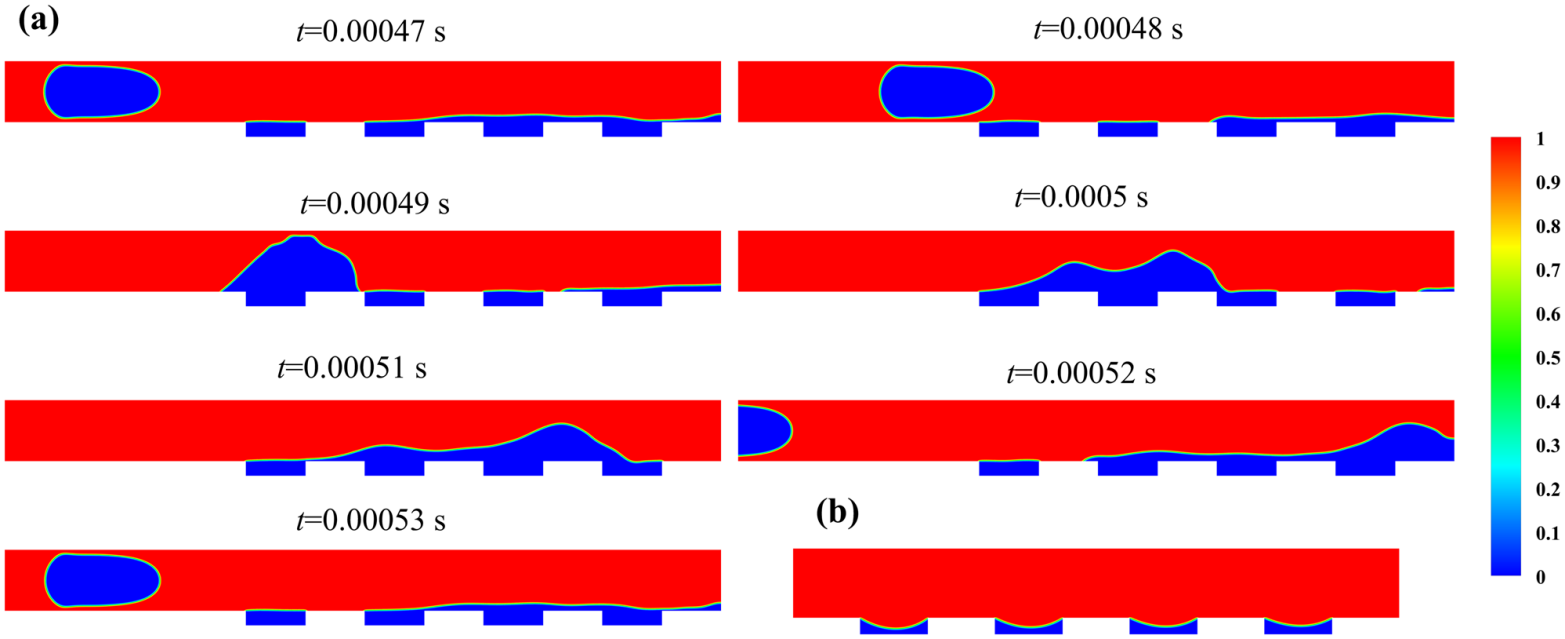
(**a**) The contours of water volume fraction for the bubbly regime at 0.00047 s until 0.00053 s, (**b**) The contour of water volume fraction for single-phase in the stable condition at 0.0002 s.

In the case of bubbly flow with a bubbles size of 3396.3 µm^2^, ${\mathrm{Re}}_{l}$ of 700, and *Ca* of 0.02 in the hydrophobic microgrooved channel, which results in the maximum pressure drop reduction, is compared with the single-phase that comes to the hydrophobic microchannel with *Re* and *Ca* of 700 and 0.02, respectively. From simulations of these cases, it is found out that the pressure drop reduction equals 70.8% for the inlet two-phase flow and 19.9% for the inlet single-phase flow. As a result, the hydrophobic surfaces work better for two-phase regimes than for single-phase flow in the optimum condition. This fact is also seen in other cases, which have a mixing procedure between bubbles and gas in microgrooves. For more sense of the mixing process and its influence on pressure drop reduction, the stable contours of water volume fraction for the time from 0.00047 s until 0.00053 s when the next repeatable period starts are depicted in Fig. 8a. Also, in Fig. 8b, the stable contour of the water volume fraction for the single-phase is displayed. From the pictures, it is evident that the mixing effect in two-phase flow leads to more performance of hydrophobic microchannel in comparison with the single-phase flow.

## Discussion

In the present study, using the phase-field method, the pressure drop reduction of bubbly flow and the controlling parameters in the hydrophobic microchannel with the rectangular microgrooves on its bottom wall were investigated. For this goal, the verification with Yamada et al*.*’s study^48^ proved the accuracy and suitability of the considered method, numbers of grids, and applied parameters. After realizing the positive impact of the hydrophobic microgrooved channel on the pressure drop reduction of bubbly flow, which was predictable based on Lewis et al*.* ’s study^37^, the effects of parameters including bubbles volume or length, the velocity of flow, the combination of these two parameters, and the surface tension between two phases were scrutinized. In essence, it was observed that raising the volume or length of the bubbles resulted in the improvement of pressure drop reduction. The more velocity of flow or Reynolds number was developed, the less pressure drop reduction increased, which is the same relationship reported for single-phase flow^51^. The combination of these two effects in the condition of mixing the bubbly flow with the air pocket in microgrooves caused a decrease in pressure drop reduction, excluding the case with ${Re}_{l}$ of 700, where the influence of bubbles length was more powerful than the flow velocity. The development of the capillary number led to the reduction of pressure drop reduction. Therefore, the optimization of conditions plays a crucial role in achieving maximum efficiency. Among the considered cases, the hydrophobic microgrooved surfaces showed interesting outcomes for the two-phase inlet flow and were even more satisfying than the inlet single-phase flow. The maximum pressure drop reduction of bubbly flow was up to 70% and almost 3.5 times higher than single-phase flow in the case with ${\mathrm{Re}}_{l}$ of 700, Ca of 0.02. This reveals the productivity of hydrophobic microgrooved surfaces for bubbly flow as Stevens et al*.*^36^ expressed. However, we could reach maximum pressure drop reduction by optimizing the effective parameters of flow regimes, as Bullee et al. 38 proved that adding more air did not necessarily lead to more drag reduction. Based on this, these surfaces can be employed for two-phase regimes in fluid transport applications in various fields such as petroleum, medicine, etc. However, the related parameters and their combination effects should be considered at the moment of use. Apart from considering flow essential parameters, the hydrophobicity effective parameters like contact angle and surface wettability are also important and can be the focus of research for future works.

## Methods

### Governing equations and solution strategy

The continuity, Navier–Stokes, and phase-field are coupled for this two-phase flow problem. Among many methods available for solving two-phase systems, the continuous method of phase-field reveals promising results in this study due to capturing the fluid interface and pressure drop more precisely and reducing the computational cost^22,52^. This free energy-based method works by defining a phase-field variable ($\phi$) that can be altered between − 1 (gas) and 1 (liquid)^53,54^. Instead of explicitly tracking the interface, this method represents the interface as a continuous transition region. The phase-field variables are discretized in both space and time, allowing the simulation to advance in time while updating these variables across the computational grid. The mentioned governing equations for immiscible and incompressible two-phase flows are as follows:

2 $$\nabla \cdot u = 0$$

3 $$\rho \frac{\partial u}{\partial t}+\rho \left(u\cdot\nabla \right)u=\nabla \cdot\left[-PI+\mu (\nabla u+({\nabla u)}^{T})\right]+G\nabla \phi $$

4 $$\frac{\partial \phi }{\partial t}+u\cdot\nabla \phi =\nabla \cdot M \nabla G$$

In Eqs. (1) and (2), $u$, $\rho$, $t$, $P$, *I*, $\mu$ and $G$ represent velocity, density, time, pressure, unit tensor, dynamic viscosity, and chemical potential. Although gas is compressible, due to the microchannel’s low flow velocity and small dimension, the assumption of incompressibility is acceptable. In the Navier–Stokes equation, the volume force within the interface region is added and modelled as $G\nabla \phi$^55^. $G$ in the surface tension force ($G\nabla \phi$) is derived from the free energy equation as:

5 $$G=\lambda \left[-{\nabla }^{2}\phi +\frac{\phi ({\phi }^{2}-1)}{{\epsilon }^{2}}\right]$$

where $\lambda$ and $\epsilon$ show the interfacial energy density and the capillary width. Capillary width controls the interface thickness and smoothly transition of flow, and these two parameters have a relationship with the surface tension coefficient ($\sigma$) as follows:

6 $$\lambda =\frac{3\epsilon \sigma }{\sqrt{8}}$$

Based on this equation, the thinner thickness of the interface results in lower interfacial energy density. Equation (3) demonstrates the advection–diffusion equation, which is known as the Cahn–Hilliard equation for predicting the interface of two-phase flow. $M$ stands for mobility, which is the diffusion component^56,57^. It is related to $\epsilon$ with *χ*, which is the mobility tunning parameter as:

7 $$M=\chi {\epsilon }^{2}$$

The $\chi$ should be large enough to create a constant interface thickness and also low enough not to add extra diffusion and not to eliminate the effect of convection terms^58^. It should be noted that the value of $\rho$ and $\mu$ in two-phase flows are defined based on $\rho$ and $\mu$ of liquid and gas (subscripts $l$ and $g$) as follows:

8 $$\rho =\frac{1+\phi }{2}{\rho }_{l}+\frac{1-\phi }{2}{\rho }_{g}$$

9 $$\mu =\frac{1+\phi }{2}{\mu }_{l}+\frac{1-\phi }{2}{\mu }_{g}$$

The Reynolds number and capillary number are described by:

10 $${Re}_{(l,g)}=\frac{{\rho }_{(l,g)}{U}_{(l,g)}{D}_{h}}{{\mu }_{(l,g)}}$$

11 $$Ca=\frac{{\mu }_{l}{U}_{l}}{\sigma }$$

where ${U}_{(l,g)}$ and ${D}_{h}$ denote the superficial liquid or gas velocity and hydraulic diameter.

The Finite Element Method (FEM) is used to solve the equations, while the Cahn–Hilliard phase-field equation is applied to model two-phase flow. Also, the standard streamwise upwind Petrov–Galerkin (SUPG) method is used for solving the Navier–Stokes equation. The weak formulations from the SUPG method are applied to the triangular mesh. Due to incompressible flow, the pressure and velocity basic functions are discretized as one order and two orders for achieving high accuracy for flow with low Reynolds number^59^. The implicit Backward Differentiation Formula method (BDF) is also utilized as a time-stepping method, and the Parallel Sparse Direct Solver (PARDISO) is implemented to determine the linear system equations.

In all the simulations, the residual tolerance and time step are 0.001 and 1 µs, respectively. Also, $\epsilon$ is set to be half of the mesh size to generate the suitable interface thickness at each domain point. Due to the importance of choosing the appropriate $\chi$ (or $M$), its rough estimation is initially calculated based on Eq. (11), which results from the scale analysis of Eq. (3). Then, since mobility is dependent on the inherent physical properties of the system, with the help of experimental data, the correct value of $\chi$ can be specified and implemented in each simulation^35^.

12 $$\chi =\frac{2\sqrt{2}{U}_{max}}{3\sigma }$$

In this equation, ${U}_{max}$ is the maximum velocity magnitude in the domain.

### Boundary condition

**Figure 9:**
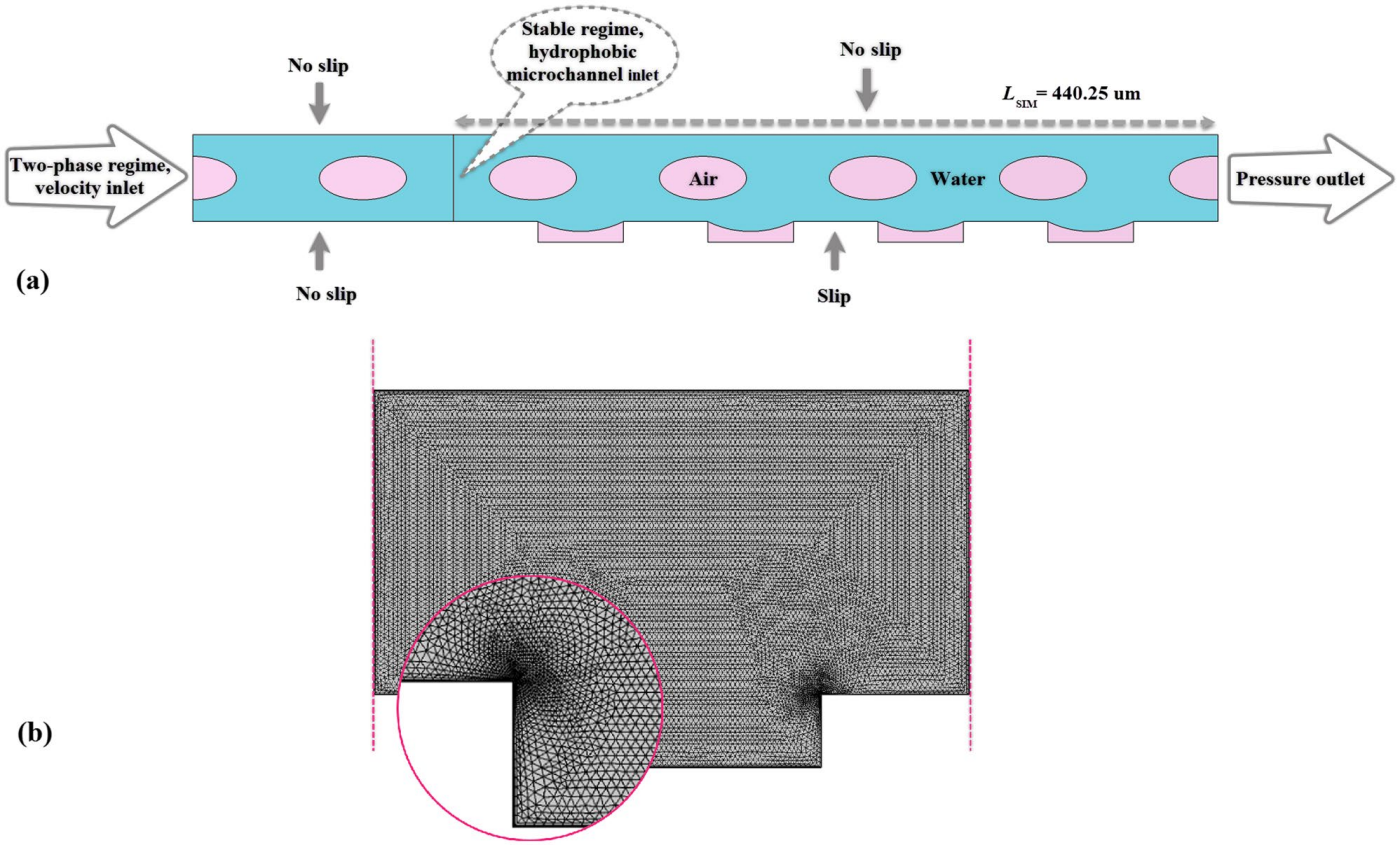
(**a**) The considered boundary conditions in the simulations. (**b**) The computational grid and a close snapshot of the corner.

The boundary conditions for the simulations and also the mesh shape for one microgroove are displayed in Fig. 9a,b, respectively.

Before bubbly flow comes into the hydrophobic microgrooved channel, it is developed and stabilized along a hydrophilic microchannel. For that purpose, a developed laminar velocity is considered at the inlet of the hydrophilic microchannel. It should be noted that the dimension of hydrophilic microchannel length should be long enough such that the bubbly regime is improved and a fixed shape is produced. The boundary condition of the outlet of the hydrophobic microchannel is the pressure-based outlet. For the upper and lower microchannel walls, the no-slip boundary condition with a contact angle and the slip boundary condition with specific slip length and contact angle are presented, respectively, which are determined by Eqs. (12–14):

13 $$n\cdot{\epsilon }^{2}\nabla \phi ={\epsilon }^{2}\mathrm{cos}\theta \left|\nabla \phi \right|$$

14 $$n\cdot M \nabla G=0$$

15 $$u=\left\{\begin{array}{c}0\, for\, top\, wall\\ {u}_{w} \,for\, bottom\, wall\end{array}\right.$$

where $n$, $\theta$, and ${u}_{w}$ indicate the unit normal vector to the wall, contact angle, which equals 140° in simulations of this study, and slip velocity, respectively. The slip length (${L}_{s}$) is measured from Eq. (15) for each simulation and is applied on the bottom wall. This equation is based on the coupled liquid–gas interface model of slip length that Woolford et al. presented for hydrophobic surfaces with microgrooved configurations for the laminar flow^60^. It depends on the microgroove sizes and velocity. In this equation, ${\mathrm{Re}}_{w}$ is the Reynolds number which is specified based on sum of width and pitch of microgroove size ($w$), $w={w}_{g}+{w}_{p}$ (see Fig. 1).

16 $${L}_{s}=\frac{{w}_{g}+{w}_{p}}{2\pi }\mathrm{ln}\left[\mathrm{sec}(\frac{\pi {w}_{g}}{2({w}_{g}+{w}_{p})})\right]\left[0.172+\frac{2.36\times {10}^{5}}{{({Re}_{w}+540)}^{2}+2.1\times {10}^{4}}\right]$$

The investigated cases are brought in Table 3, and they are distinguished based on the Reynolds number of liquid in this study, and the velocity of water and air is the same in each case.

**Table 3:** The considered cases in this study.

${\mathrm{Re}}_{l}$	${U}_{l} (\mathrm{m}/\mathrm{s})$	${U}_{g}(\mathrm{m}/\mathrm{s})$	${L}_{s}(\mathrm{\mu m})$
140	1.5	1.5	3.57
361	3.8	3.8	2.46
535	5.6	5.6	2.02
700	7.4	7.4	1.74
879	9.3	9.3	1.55

## Data Availability

All data generated or analyzed during this study are included in this article; other additional datasets used during the current study are available from the corresponding author on reasonable. request.
